# Inter-Item Differences in Metacognitive Judgments: Insights into the Collective Wisdomware Underlying These Judgments

**DOI:** 10.3390/bs16071103

**Published:** 2026-07-02

**Authors:** Asher Koriat, Noam Yehudai

**Affiliations:** 1 Department of Psychology, University of Haifa, 199 Abba Hushi Ave., Haifa 3103301, Israel; 2 Institute of Information Processing and Decision Making, University of Haifa, 199 Abba Hushi Ave., Haifa 3103301, Israel; nyehudai@staff.haifa.ac.il

**Keywords:** metacognitive judgments, inter-item differences, collective wisdomware

## Abstract

Previous research introduced the concept of collective wisdomware within the frame-work of the self-consistency model of subjective confidence. According to this model, confidence in a binary choice arises from the random sampling of cues from a collective pool that is shared by individuals with similar backgrounds. Consequently, confidence should increase with the degree of agreement among respondents—a consensuality effect—and should predict the likelihood that other respondents will make the same choice—a replicability effect. This framework has recently been extended to judgments of learning (JOLs) and feelings of knowing (FOKs), and both types of judgments have likewise been shown to exhibit consensuality and replicability effects. In the present study, we report findings based on a reanalysis of previous data for confidence judgments, JOLs, and FOKs, indicating that the assumption of a uniformly shared collective wisdomware does not hold equally across all items. We consolidated data from several samples, including university students and school children, who completed computerized memory and perceptual tasks. Metacognitive judgments (confidence, FOK, JOL) were collected and analyzed for consensuality and replicability across and within participants. For each type of judgment, we identify reliable inter-item differences in the magnitude of the consensuality and replicability effects and examine several correlates of these item-level variations. Split-half reliability analyses confirm that this variance is a stable property of the items. The goal is to shed light on the nature of the collective wisdomware underlying each form of metacognitive judgment, as well as across these judgments. Our findings demonstrate that reliance on shared cues depends heavily on specific item characteristics.

## 1. Introduction

Much of the work on confidence judgments has emphasized the accuracy of these judgments. However, research conducted mostly in our lab has shown that confidence judgments actually track the consensuality of the choice—the popularity of that choice among participants. In that research, two-alternative forced-choice questions were used in several domains. For each question, participants chose the correct answer and indicated their confidence in the correctness of that answer. In all studies, a deliberate attempt was made to include a sufficiently large number of items for which the popular answer is the wrong answer.

The results supported the consensuality principle (see Koriat, 2008a, 2012, 2018): Confidence increased with the consensuality of the choice so that the confidence/accuracy correlation was positive for consensually correct (CC) items but negative for consensually wrong (CW) items. This pattern of results has been observed across a wide range of domains.

The self-consistency model was proposed to account for these results. The model assumes that when facing a binary choice task, participants retrieve a number of item-specific cues. They choose the option supported by the majority of these cues, and their confidence in the correctness of their choice is based on the consistency with which the chosen option was supported across the retrieved cues. It was further assumed that the cues are retrieved randomly from a pool of item-specific cues that is shared by participants with similar backgrounds. This pool—the cues and their implications for the choice in question—was termed collective wisdomware. Simulation experiments that embodied these assumptions yielded results that captured closely the consensuality effect that has been observed across several domains.

Related to the consensuality effect is the replicability effect. Because participants are assumed to sample their cues for each item largely from the same wisdomware, the confidence with which a specific option is endorsed was expected to predict the likelihood that other participants will choose the same option. This prediction was confirmed for nine different domains (Koriat, 2025).

The consensuality and replicability effects were also observed for items for which the choice does not have a truth value (e.g., personal beliefs and attitudes, and personal preferences, see Koriat, 2025). This result was taken to suggest that people’s confidence in a choice reflects the likelihood that the choice is reliable, likely to be made again in the future. Therefore, the proper performance criterion for confidence judgments is not the accuracy of these judgments but their reliability and replicability. It was proposed that future studies should report Confidence/Replicability (Conf/Rep) correlations along with the confidence/accuracy correlations (Koriat, 2025).

The self-consistency framework for subjective confidence has been generalized recently to feelings of knowing (FOKs) and to judgments of learning (JOLs) (see Koriat, 2026). To do so, two scores were derived from FOKs and JOLs, which correspond to choice and confidence in studies of confidence judgments. Thus, FOK Choice represented the categorical distinction between positive and negative FOKs, whereas FOK Strength represented the strength of the respective FOK-Choice response. The results yielded clear evidence for the consensuality effect: the FOK Strength associated with a FOK-Choice response increased significantly with the consensuality of that response. In addition, differences in the FOK Strength associated with a FOK-Choice response were positively correlated with differences in the likelihood that other participants will make the same FOK-Choice response.

A similar analysis was applied to JOLs. Because JOLs were elicited on a 0–100 scale, representing the likelihood of recalling the target at test, the JOL-Choice response represented the categorical distinction between a positive JOL (50% or over) and a negative JOL (below 50%), whereas JOL Strength represented the strength of the respective JOL-Choice response. The results also yielded clear evidence for the consensuality and replicability effects: the JOL Strength associated with a JOL-Choice response increased with the consensuality of that response and predicted the likelihood that others would make the same JOL-Choice response. Thus, confidence, FOK judgments, and JOLs seem to entail a similar process: retrieving item-specific cues randomly from collective wisdomware.

In this article, we report on findings for confidence, FOKs, and JOLs that may provide some insight into the nature of the collective wisdomware assumed to underlie these judgments. So far, little is known about the shared cues that people with similar backgrounds rely on in making their first-order and second-order judgments. However, unexpected results on JOLs, reviewed here in Study 1, suggested that the assumption of reliance on a shared pool of cues does not apply to all items alike.

### Research Questions and Hypotheses

The primary aim of this article is to investigate inter-item differences in metacognitive judgments and their reliance on collectively shared cues. The present secondary analysis addresses the following research questions and hypotheses:

1. Does the reliance on collective wisdomware vary significantly across items? We hypothesize reliable inter-item differences in the magnitude of the consensuality and replicability effects for JOLs, FOKs, and confidence.
2. Are these inter-item differences stable properties of the stimuli rather than statistical noise? We hypothesize that split-half analyses will confirm the reliability of these differences across independent samples.
3. How do specific item properties (e.g., semantic relatedness, information accessibility) predict the degree of reliance on shared cues? We hypothesize that related items and those eliciting high accessibility will show significantly greater cross-individual replicability.

## 2. Materials and Methods

The present article is based on the comprehensive reanalysis of previously published datasets from several experimental studies conducted by our research group. Detailed methodologies for each specific study dataset are described in the respective original publications (e.g., Koriat, 1995, 2011; Koriat & Bjork, 2005; Koriat et al., 2006).

### 2.1. Participants

The aggregated sample encompasses several hundred participants across the included studies. The majority of participants were undergraduate university students. Participants typically completed the experiments in exchange for course credit or monetary compensation.

### 2.2. Materials

The materials varied depending on the specific metacognitive judgment being assessed. JOL tasks utilized lists of paired associates (ranging from 60 to 108 pairs per list) categorized into a priori association, a posteriori association, and unrelated pairs based on free-association norms. FOK tasks utilized 95 general-knowledge questions requiring a one- or two-word answer. Perceptual tasks utilized 40 pairs of irregular lines and 40 pairs of geometric shapes.

### 2.3. Procedure

All data collection procedures were computerized. Participants were required to provide a metacognitive judgment (JOLs, FOK, or confidence) following a choice or a retrieval attempt. For JOL tasks, participants were presented with cue-target pairs and rated the likelihood of recalling the target on a 0–100 scale. For FOK tasks, participants attempted to answer general-knowledge questions, provided FOK judgments for unrecalled items, and then completed a recognition test. For perceptual tasks, participants made two-alternative forced-choice decisions followed by confidence ratings on a 0–100 scale.

### 2.4. Analytical Methods

Continuous metacognitive judgments (0–100 scales) were dichotomized to derive two metrics: Choice (a categorical variable indicating a positive vs. negative judgment) and Strength (the absolute magnitude associated with that choice). Consensuality was evaluated via *t*-tests comparing the mean judgment strength for consensual versus nonconsensual responses. Replicability (cross-individual) was indexed by the Pearson correlation (*r*) computed across items between a participant’s judgment strength and the percentage of other participants making the same choice. Replicability (within-individual) was indexed by point-biserial correlations across repeated presentations. Split-half reliability was used to determine the stability of item properties by splitting participants into two groups and correlating their replicability scores across items. For tests where indices of variance and effect sizes were not originally reported, effect sizes (Cohen’s *d*) and exact *p*-values were mathematically derived from the available test statistics (*t*) and degrees of freedom using standard conversion formulas (Cohen, 1988).

### 2.5. Ethical Considerations

Ethical review and approval, as well as patient consent, were waived for this study, as it is based exclusively on the reanalysis of the results of previously published studies that had received ethical approval.

## 3. Results

### 3.1. Study 1: Judgments of Learning for Related and Unrelated Paired Associates

Study 1 summarizes and elaborates on the results reported in Koriat (2026) that yielded the unexpected findings about inter-item differences in the collective cues underlying JOLs. These results were obtained in Studies 2 and 3 of that study.

Study 2 in Koriat (2026) was based on a reanalysis of the results of Experiment 1 of Koriat and Bjork (2005). In that experiment, 24 participants were presented with a list of 60 paired associates, 20 with high associative strength, 20 with low associative strength, and 20 unrelated pairs. The average association strength for the three classes of items was 0.564, 0.065, and 0, respectively. In Table 1, we present results on differences between the three classes of items in the consensuality effect, using participants for whom there was variance in the JOL-Choice response and in JOL Strength. It can be seen that the consensuality effect was highly significant for the low-association and the high-association pairs, but not for the unrelated pairs.

As expected, the results for the replicability effect yielded a similar pattern. The replicability effect was indexed by the JOL/Rep correlation—the Pearson correlation calculated for each item between the JOL Strength associated with a JOL-Choice response and the percentage of other respondents who made the same JOL-Choice response. As reported in Koriat (2026), the mean JOL/Rep correlation across items was highly significant for both the high-association pairs, *r* = 0.48, *t*(23) = 10.55, *p* < 0.001, *d* = 2.15, and the low-association pairs, *r* = 0.41, *t*(23) = 7.82, *p* < 0.001, *d* = 1.60, but was not significant for the unrelated pairs, *r* = 0.04, *t*(23) = 0.57, *p* = 0.576, *d* = 0.12. These results suggest that people share the cues underlying JOLs for the related pairs but do not share these cues for the unrelated pairs.

Other results reported in Koriat (2026) were based on a reanalysis of the data from Koriat et al. (2009), which concerned JOLs made by school children aged 7 to 12. The analyses yielded robust consensuality and replicability effects for the related paired associates. Although these effects were significant for both the related and unrelated pairs, they were significantly stronger for the related pairs than for the unrelated pairs.

### 3.2. Study 2: FOK Judgments

Study 2 concerns FOK judgments. The results are based on a reanalysis of the results of Koriat (1995). Here, we focus on inter-item differences. We first examine the reliability of item differences in the magnitude of the replicability effect for FOK judgments. We then examine the correlates of these differences.

In that study, 95 general-knowledge questions in Hebrew were presented to 36 Israeli participants, first in an open-ended format (recall), and then in a four-alternative forced-choice format (recognition). All items required a one-word or a two-word answer, which participants attempted to recall. They then made FOK judgments whether they had supplied an answer or had not.

Two scores were derived from each reported FOK, FOK Choice, and FOK Strength. In Koriat (2026), a Pearson correlation was calculated between the FOK strength associated with a FOK-Choice response and the percentage of other participants who made the same FOK-choice response. That correlation, labeled FOK/Rep correlation, averaged 0.22 across 90 items and was highly significant, *t*(89) = 7.55, *p* < 0.001, *d* = 0.80. This result suggests that participants generally rely on collectively shared cues in making their FOK judgments. In this study, we used a split-half procedure to assess the reliability of inter-item differences in the replicability effect for FOK judgments. Participants were divided into two groups, even-numbered and odd-numbered. For each participant in each group, the FOK/Rep correlation was calculated for each item. The correlation across items between the two FOK/Rep correlations was calculated across 84 items for which there was variance in the FOK-Choice response and in FOK Strength. That correlation was *r* = 0.53, which was highly significant, *t*(83) = 5.65, *p* < 0.001, *d* = 0.62.

These results suggest that although FOK judgments generally rely on collectively shared cues, there are significant inter-item differences in that reliance. As noted earlier, the mean FOK/Rep correlation across all participants was 0.22, significantly higher than 0. However, the mean FOK/Rep correlation across participants ranged from −0.41 to +0.90 across items. A mean FOK/Rep correlation of 0.21 is significant at the 0.05 level. For 45 items, mean FOK/Rep was higher, whereas for the remaining 45 items, it was at or below this level.

To address why this conclusion relies on the mean correlation rather than its variance, and how the reliability analysis pertains to this conclusion, the mean value (0.22) confirms the overall presence of a collective cue effect across the task. However, the wide variance (from −0.41 to +0.90) could simply be statistical noise. The highly significant split-half reliability correlation (*r* = 0.53) demonstrates that this variance is not random noise, but rather a stable, intrinsic property of the specific questions themselves. Thus, the tendency to pull from the collective wisdomware varies reliably between items.

To gain some insight into these item differences, we calculated the correlations across all 90 items between the mean FOK/Rep correlation for each item and the means of several item properties that had been used in Koriat (1995). These correlations appear in Table 2.

The first observation to notice in Table 2 is the high correlation (0.81) between accessibility (ACC) and mean FOK. ACC represents the number of participants who produced a response in the recall part of the study, a correct answer, a commission error, or a partial correct or incorrect answer. The correlation between ACC and mean FOK is consistent with the accessibility hypothesis of FOK (Koriat, 1993), according to which FOK judgments are based on the amount of partial information that comes to mind when attempting to recall the answer rather than on direct access to stored memory traces (see Schwartz, 2024).

In turn, ACC and mean FOK yielded the highest correlations with mean FOK/Rep. These correlations suggest that the FOK items that yield the strongest evidence for reliance on collective wisdomware are those that tend to bring to mind a large amount of full or partial information during attempted recall, regardless of the accuracy of that information.

Mean FOK/Rep was also correlated significantly with RCG (mean correct recognition). This correlation, however, may reflect in part the observation that most of the information that comes to mind tends to be correct (Koriat, 1993; Koriat & Goldsmith, 1996). Indeed, when the contribution of ACC to mean FOK/Rep was partialled out, the correlation between RCG and mean FOK/Rep was *r* = 0.19, *t*(87) = 1.79, *p* = 0.077, *d* = 0.19.

In sum, the results suggest that the self-consistency framework, when applied to FOK judgments, finds strong support for items that bring to mind many cues regardless of their accuracy. Possibly, these items also produce many fleeting cues that are not readily reportable. These results are consistent with the finding reported earlier that the replicability effect for JOLs is stronger for related paired associates than for unrelated pairs. Possibly unrelated pairs tend to bring to mind fewer cues than related pairs, and most of these cues are idiosyncratic rather than collectively shared.

### 3.3. Study 3: Judgments of Learning: Replicability Within Individuals and Across Individuals for a Priori, a Posteriori, and Unrelated Paired Associates

The analyses presented in this study took advantage of the results of Experiment 1 of Koriat et al. (2006), which concerned JOLs. Because the experiment included two study-test cycles of the same list, the results allow assessment of the replicability effect both within individuals across repeated presentations of the same items, and also across individuals. In addition, they help determine the type of paired associates for which JOLs tend to be based on collectively shared cues.

In Experiment 1 of Koriat et al. (2006), 40 participants were presented with a list of 72 paired associates in Hebrew. The list included 24 pairs with high a priori association, 24 purely a posteriori pairs, and 24 unrelated pairs. A priori association refers to the probability with which the cue word, when presented alone, brings to mind the target word (as reflected in word association norms). In contrast, a posteriori association refers to the perceived association between the cue and the target when both are present. For these pairs, neither word is an a priori associate of the other word, but the two words appear related when presented together (e.g., bed-night, see also Fischler, 1977).

The study as a whole was intended to test hypotheses about the Underconfidence-with-Practice Effect (Koriat et al., 2002), but the analyses to be presented here focused on inter-item differences in JOLs. The results were analyzed in the same way described earlier, deriving a JOL-Choice response and a JOL Strength from each reported JOL.

Using participants who had both consensual and nonconsensual means, the average JOL Strength for consensual and nonconsensual JOL-Choice responses was calculated for each participant, and the means across participants are presented in Table 3.

It can be seen that the consensuality effect was significant for each of the three classes of items. However, consistent with previous results, the magnitude of the consensuality effect for the unrelated pairs was smaller than that for the a priori pairs, *t*(19) = 2.80; *p* = 0.011, *d* = 0.63, and also than that for the a posteriori pairs, *t*(19) = 2.28; *p* = 0.034, *d* = 0.51. What is quite instructive, however, is that the consensuality effect was significant even for the a posteriori pairs, for which the normative association between the two words is zero. Although the consensuality effect was somewhat weaker for the a posteriori pairs than for the a priori pairs, the difference was not significant, *t*(31) = 1.73, *p* = 0.093, *d* = 0.31.

Turning next to the replicability effect, two types of replicability indexes were calculated. The first is the within-individual JOL/Rep correlation: the point-biserial correlation across items between the JOL Strength associated with a JOL-Choice response in Presentation 1 and the likelihood of making the same JOL-Choice response in Presentation 2. This correlation taps the degree to which the ensemble of cues underlying JOLs for each item in Presentation 2 overlaps with that underlying JOLs in Presentation 1. The second is the cross-individual JOL/Rep correlation as that used in Study 1: The Pearson correlation between the JOL Strength associated with a JOL-Choice response and the percentage of other participants who made the same JOL-Choice response. These JOL/Rep correlations were calculated separately for each presentation and for each class of items. The average correlation coefficients after applying Fisher’s z-transformation appear in Table 4.

Two observations should be made about the results. The first is that although the cross-individual replicability effect was not significant for the unrelated pairs, these pairs did yield a significant within-individual replicability effect, indicating some consistency in the cues used by the same person across the two presentations. These results suggest that in responding to unrelated items, people rely on idiosyncratic cues but tend to use the same idiosyncratic cues across repeated presentations.

The second observation is that the cross-individual replicability correlations were consistently higher than the respective within-individual correlations. This pattern of results is somewhat surprising, suggesting that an individual’s JOL strength predicts better other participants’ JOL-Choice responses than one’s own Choice responses to a repeated presentation of the same items. However, this difference likely stems from the reduced range (variance restriction) for within-participant correlations. Indeed, it was observed only when participants’ JOL Strength was used to predict the collective choice—the aggregated percentage of others who made the same choice, but not when it was used to predict the individual choices aggregated across all other participants.

In the latter analysis, for each item, a dichotomous same-choice score was generated by comparing the choice made by each target participant to that made by each of the other participants. A point-biserial JOL/Rep correlation was then calculated for each target participant between the JOL Strength and the dichotomous replicability score, and for each target participant, that correlation was then averaged across all other participants. For Presentation 1, this analysis yielded the following means calculated across all target participants: 0.29 for the a priori items, 0.20 for the a posteriori items, and 0.04 for the unrelated pairs.

We also examined the reliability of inter-item differences. In the first analysis, we examined the reliability of the cross-individual replicability effect in Presentation 1 using the same split-half analysis as that performed in Study 2 for FOK judgments. The correlation across items between the JOL/Rep correlations for the two groups was calculated across items for which both means were available. It was *r* = 0.47, *t*(54) = 3.87, *p* < 0.001. Note that the reliability of item differences in the cross-individual replicability effect was significant when computed separately for the a priori pairs, *r* = 0.64, *t*(19) = 3.65, *p* = 0.001, but not for the a posteriori pairs, *r* = −0.06, *t*(21) = 0.28, *p* = 0.782, or for the unrelated pairs, *r* = 0.41, *t*(10) = 1.41, *p* = 0.189.

In a second analysis, we calculated the correlation across all items between the within-individual JOL/Rep correlation across the two presentations and the cross-individual JOL/Rep correlation in Presentation 1. The correlation was *r* = 0.32, *t*(70) = 2.81, *p* = 0.006. This result suggests that inter-item differences in the replicability effect are reliable whether they are evaluated within individuals or across individuals. Note, however, that this correlation too differed for different pair types: It was 0.45, *t*(22) = 2.38, *p* = 0.026, across the a priori pairs, 0.64, *t*(22) = 3.92, *p* < 0.001, across the a posteriori pairs, and only 0.07, *t*(22) = 0.31, *p* = 0.759, across the unrelated pairs.

### 3.4. Study 4: Judgments of Learning: Replicability Within Individuals and Across Individuals for Forward, Backward, and Unrelated Paired Associates

The finding in Study 3, that the consensuality and replicability effects were significant for the a posteriori pairs, suggested the possibility that these effects are also significant for Backward-associated pairs for which the association is from the target word to the cue word (e.g., cheese-mouse, see Maxwell & Huff, 2021). This possibility was examined for the results of Experiment 2 of Koriat et al. (2006), which included JOL data for Backward-associated pairs.

In that experiment, participants studied 108 paired associates. These included 72 pairs with a unidirectional association, 36 of them with a Forward association, and 36 with a Backward association. In addition, there were 36 unrelated pairs. For half of the pairs in each class, JOLs were elicited immediately after study, and for the others, JOLs were delayed (for further details see Koriat et al., 2006). Here, we examine the results only for the immediate condition in the first two presentations of the list. These results were analyzed by deriving a JOL-Choice response and a JOL Strength from each reported JOL, as was done in Study 3.

The results were analyzed in the same way as in Study 3. The first analysis was based on the results for Presentation 1. JOL-Choice responses were classified as consensual or nonconsensual according to their frequency across participants. For participants who had valid means, the average JOL Strength for consensual and nonconsensual JOL-Choice responses was calculated for each participant, and the means across participants are presented in Table 5.

The results presented in Table 5 replicate the observation that the unrelated pairs yield little evidence for a consensuality effect. In contrast, a significant consensuality effect was demonstrated for the Forward and Backward pairs. Thus, the Backward pairs yielded a significant consensuality effect, similar to what had been found for the a posteriori pairs in Study 3. Note that the consensuality effect for the Backward pairs was significantly stronger than that for the Unrelated pairs, *t*(26) = 3.84, *p* < 0.001, *d* = 0.75, but was still weaker than that observed for the Forward pairs, *t*(24) = 3.02, *p* = 0.006, *d* = 0.62.

Turning next to the replicability effect, the within-individual JOL/Rep correlation was assessed by the point-biserial correlation across items between the JOL Strength associated with a JOL-Choice response in Presentation 1 and the likelihood of making the same JOL-Choice response in Presentation 2. In turn, the cross-individual JOL/Rep correlation was assessed by the Pearson correlation across items between the JOL Strength associated with a JOL-Choice response and the percentage of other participants who made the same choice response to that item. These correlations were calculated separately for each presentation and for each class of items. The average correlations across participants, after using Fisher’s z-transformation, appear in Table 6.

The results presented in Table 6 were also similar to those of Study 3 (Table 4). First, the cross-individual replicability effect was not significant for the unrelated pairs. In contrast, the within-individual replicability effect was significant even for the unrelated pairs. As in Study 3, these results suggest that participants rely on idiosyncratic cues in making JOLs for unrelated pairs but tend to use the same cues consistently across the two presentations.

Second, focusing on the Forward and Backward pairs, the cross-individual JOL/Rep correlations for these pairs were consistently higher than the respective within-individual correlations, suggesting that for each individual, the JOL strength associated with a JOL-Choice response predicts better the JOL-Choice responses of other participants than one’s own future choice responses. However, as in Study 3, we found that this was true only when JOL Strength was used to predict the percentage of others who made the same JOL Choice, but not when JOL Strength was used to predict the individual JOL Choices of other participants, possibly due to range restriction constraints. Thus, a similar analysis to that used in Study 3 was applied to the results for Presentation 1. It yielded the following JOL/Rep means averaged across all target participants: 0.44 for the Forward pairs, 0.38 for the Backward pairs, and 0.04 for the unrelated pairs.

We also assessed the reliability of inter-item differences by dividing participants into two groups and calculating the JOL/Rep correlation for each group. Somewhat surprisingly, the correlation between the JOL/Rep correlations for the two groups across items was *r* = 0.16, *t*(85) = 1.47, *p* = 0.145. In contrast, the correlation across items between the within-individual JOL/Rep correlation for each item and the cross-individual JOL/Rep correlation in Presentation 1 was highly significant, *r* = 0.47, *t*(139) = 6.24, *p* < 0.001. When the correlation was calculated separately for each pair class, it was *r* = 0.49, *t*(47) = 3.87, *p* < 0.001, for the Forward pairs, *r* = 0.46, *t*(58) = 3.95, *p* < 0.001, for the Backward pairs, and *r* = −0.01, *t*(30) = 0.05, *p* = 0.960, for the unrelated pairs.

### 3.5. Study 5: Confidence in Perceptual Decisions

Study 5 focused on subjective confidence in tasks that tap perceptual judgments. Two such tasks have been used in several studies (e.g., Koriat, 2011, 2017; Litvinova et al., 2020). The analyses of inter-item differences to be presented here were based on the results obtained in Koriat (2011). In the Lines task (Experiment 1), participants were presented with 40 pairs of irregular lines. For each pair, they decided which of the two lines was longer. In the Shapes task (Experiment 2), they decided which of two shapes had a larger area. In both tasks, they also indicated their confidence on a 0–100 scale representing the likelihood that their choice was correct.

In the first analysis, we examined the within-individual and cross-individual replicability effects for the two tasks for the results of Koriat (2011). In that study, each of the two tasks was presented to participants five times. Here, however, we use only the results for the first two presentations in order to apply the same analysis as that used in Studies 3 and 4. The results are presented in Table 7 in the same way as in Table 4 and Table 6.

The results yielded significant within-individual and cross-individual replicability effects for both the Lines task and the Shapes task. The results for the cross-individual replicability effect suggest that even for perceptual tasks, participants’ choices and confidence are based on cues that are shared by different respondents. Note, however, that the magnitude of the cross-individual replicability effect was more moderate than that observed for JOLs in Studies 3 and 4. In addition, the difference between the magnitude of the cross-individual replicability effect and that of the within-individual replicability effect was not as big as that found for JOLs in Studies 3 and 4.

We examined the reliability of item differences in the two perceptual tasks. In the first analysis, participants were divided into two groups, and for each participant in each group, the Conf/Rep correlation was calculated for each item. The correlation across items between the two Conf/Rep correlations (across 32 items for which there was variance in choice and confidence) was not significant, *r* = 0.12, *t*(30) = 0.67, *p* = 0.508, for the Lines task, but was significant, *r* = 0.42, *t*(30) = 2.57, *p* = 0.015 for the Shapes task.

In a second analysis, we calculated the correlation across items between the within-individual Conf/Rep correlation for each item (across the first two presentations) and the cross-individual Conf/Rep correlation in Presentation 1. The correlation was *r* = 0.41 for the Lines task, *t*(36) = 2.68, *p* = 0.011, and *r* = 0.52, *t*(33) = 3.54, *p* = 0.001, for the Shapes task.

Additional support for the reliability of the item differences in the two perceptual tasks comes from a recent study in which these tasks were performed by Israeli and Japanese participants (Koriat & Yehudai, 2026). The correlation across items between the Conf/Rep correlations for Israel and those for Japan was significant for both the Lines task (*r* = 0.33) and the Shapes task (*r* = 0.44). Thus, the perceptual stimuli differed reliably in the extent to which participants’ judgments relied on cues that are shared by members of one’s own culture and also by those shared by members of the other culture. Additional results indicated that the reliance on collectively shared cues is stronger for items for which the choice is endorsed with higher confidence and with shorter response times.

## 4. Discussion

In what follows, we first comment on the concept of collective wisdomware. Focusing then on inter-item differences in the replicability effect, we review the results on the reliability of these differences for different forms of metacognitive judgments. We then examine the item characteristics that distinguish between the items for which participants’ judgments suggest stronger reliance versus weaker reliance on collectively shared cues. Finally, we discuss the implications of this study for the self-consistency framework.

### 4.1. The Concept of Collective Wisdomware and Broader Metacognitive Literature

The concept of collective wisdomware is a hypothetical construct (MacCorquodale & Meehl, 1948). It was invoked to account for the observation that confidence judgments in binary tasks track the consensuality of the selected option rather than its accuracy. Because the consensuality effect has been observed across several domains, the notion of collective wisdomware was assumed to apply to each of these domains. For each domain, people with similar backgrounds are assumed to share the cues underlying their first-order and second-order judgments as well as the implications of these cues for the choice in question. Beyond self-consistency, broader metacognitive research supports the notion that metacognitive judgments rely on accessible, experience-based cues. For instance, studies examining cue-familiarity heuristics in FOK tasks demonstrate that judgments are strongly influenced by the familiarity of the target cue, regardless of eventual accuracy (Metcalfe et al., 1993; Reder, 1987; see Schwartz, 2024). Similarly, contemporary research into JOLs posits that judgments are often based on perceptual and processing fluency, as well as pre-existing beliefs about how certain item manipulations will impact memory (Dunlosky & Metcalfe, 2009; Rhodes & Castel, 2008).

Many of these cues are not accessible to introspection and report. In addition, research has indicated that asking people to list the reasons for their choices can sometimes interfere with the natural decision process by leading participants to focus on verbalizable attributes (McMackin & Slovic, 2000; Wilson & Schooler, 1991). Therefore, the information about the nature of the collectively shared cues must rest on indirect inferences from a variety of observations.

In this study, we attempted to gain some insight into the collective wisdomware assumed to underlie metacognitive judgments by examining inter-item differences in the replicability effect across individuals and within individuals. We took advantage of the observation (Koriat, 2026) that the self-consistency theoretical framework applies not only to confidence judgments but also to FOK judgments and JOLs. This observation allowed us to examine results for the three forms of metacognitive judgments to gain some insight into the differences between items that are more likely or less likely to recruit cues that are shared by other participants. Of course, this pooling does not imply that the same specific cues underlie different forms of metacognitive judgments (but see Pournaghdali et al., 2025).

As noted in Koriat (2026), the focus on inter-item differences in metacognitive judgments is consistent with the item-based methodology in metacognition (Koriat, 2008a, 1995). This methodology, which focuses on item differences in metacognitive judgments, has yielded valuable information about these judgments (Brewer & Sampaio, 2006; Brewer et al., 2005; Koriat & Bjork, 2005, 2006; Koriat & Lieblich, 1977; Sampaio et al., 2018). For example, binary general-knowledge questions were found to differ reliably in confidence independent of which answer was selected (Koriat, 2008b). Results suggested that this is because items differ in the domain familiarity of the question and in the tendency of the question to bring to mind either few or many thoughts. It was proposed that selective focusing on supportive evidence should yield inter-item differences in confidence independent of which option is selected. However, the studies that relied on the item-based methodology did not explore the implications of inter-item differences for the collective wisdomware underlying metacognitive judgments.

The assumption that individuals draw on a shared pool of cues to make judgments resonates with several models in judgment and decision-making. The Lens Model of Brunswik (1952) posits that individuals make inferences based on probabilistic cues available in their ecological environment, and therefore inevitably rely on a common foundation. Gigerenzer et al. (1991) proposed that subjective confidence is derived from a ‘reference class’ of cues shared within a person’s natural environment. This was taken to explain why human heuristics are generally successful (Todd & Gigerenzer, 2012). Fiedler et al. (2023), on the other hand, argued that the systematic constraints inherent in the natural ecology from which people draw their samples can account for some of the common errors that participants make. Galesic et al. (2012, 2018) obtained evidence suggesting that people infer social consensus by sampling instances from their immediate, shared social environments. These results gave rise to their Social Sampling Model (see also Krueger et al., 2012). Together, these frameworks provide a robust theoretical foundation for the concept of collective wisdomware, illustrating how shared environmental and social cues lead to high cross-individual replicability.

### 4.2. The Reliability of Item Differences in the Reliance on Collectively Shared Cues

The most general observation reported in this study concerns the reliability of inter-item differences in the magnitude of the cross-individual replicability effect. This effect was assumed to tap the extent to which different people rely on a shared pool of cues in making their judgments.

In Study 2 on FOK judgments, participants were divided into two groups, and the magnitude of the FOK/Rep correlation for each item was found to be highly reliable across the two groups. With regard to JOLs, a similar split-half procedure applied to the results of Study 3 yielded reliable item differences in the cross-individual replicability effect. This effect was significant when calculated separately across the a priori pairs but not across the a posteriori pairs. In Study 4, in contrast, the reliability of the cross-individual replicability effect was significant when calculated across all items and also when calculated separately across the Forward or the Backward pairs. Note that in none of the analyses of JOLs did the results for the unrelated paired associates yield reliable item differences in cross-individual replicability.

Study 5 concerned confidence in two perceptual tasks (Koriat, 2011). The Conf/Rep correlation across items was not reliable across participants for the Lines task, but was reliable for the Shapes task. In turn, the correlation across items between the within-individual Conf/Rep correlation for each item (across the first two presentations) and the cross-individual Conf/Rep correlation in Presentation 1 was reliable for both tasks. In addition, in another study (Koriat & Yehudai, 2026) in which these perceptual tasks were presented to participants in Israel and Japan, the results suggested reliable inter-item differences in the extent to which participants’ confidence judgments relied on cues that are shared by members of one’s own culture as well as those shared by members of the other culture.

Altogether, the results suggest that the reliability of item differences in the magnitude of the replicability effect is quite general. This reliability was found for different forms of metacognitive judgments—FOK, JOL, and confidence. It was also demonstrated for verbal materials as well as visual stimuli.

### 4.3. The Within-Individual and Cross-Individual Replicability Effects

The reliability of inter-item differences was also supported by the results that compared the magnitude of the replicability effect within individuals to that observed across individuals. In Studies 3, 4, and 5, the replicability effect was assessed within individuals across two presentations of a list of items and also across individuals for the first presentation. The magnitude of the within-individual replicability effect for each item was found to correlate significantly across items with the magnitude of the cross-individual replicability effect. In Study 5, this was true for both the Lines task and the Shapes task. These results indicate that the same item differences in the replicability effect occur within an individual across two presentations of the same items and across individuals for each presentation of these items.

However, both Studies 3 and 4 yielded an interesting difference between the two types of replicability effect. Unlike the cross-individual replicability effect, which was not significant for the unrelated paired associates, the within-individual replicability effect was significant even for these pairs. This pattern of results suggests that although participants rely on idiosyncratic cues in responding to the unrelated paired associates, they tend to use the same cues consistently across repeated presentations of the same item. These results have implications for the cue-replication hypothesis as discussed below. As noted in the results, when uncorrected, cross-individual correlations appeared higher than within-individual correlations. However, this is largely a statistical artifact of reduced variance. Individuals tend to be highly consistent with themselves, which restricts the variance available for within-participant correlations and thus attenuates the effect size relative to tests that aggregate group-level predictions.

### 4.4. Distinguishing Between Items That Evidence Weaker vs. Stronger Reliance on Collectively Shared Cues

The results summarized above motivated examination of the differences between the items for which metacognitive judgments evidence stronger reliance vs. weaker reliance on collectively shared cues. The strongest inter-item difference observed so far is that between related and unrelated paired associates for JOLs. This difference was observed when relatedness was defined in terms of the cue-to-target associative strength according to word association norms. In Study 1, related pairs yielded a robust cross-individual replicability effect, whereas unrelated pairs evidenced no sign of a replicability effect. This was also the case in Study 3 in comparing a priori pairs with unrelated pairs, and also in Study 4 in comparing the Forward pairs with the Unrelated pairs. The results for school children (see Koriat, 2026) yielded significant replicability effects for both related and unrelated paired associates, but these effects were significantly stronger for the related pairs than for the unrelated pairs. Altogether, the results suggest that the JOLs for unrelated paired associates draw much less heavily on a collectively shared pool of cues than the JOLs for related paired associates.

For FOK judgments (Study 2), the results indicated that reliance on collectively shared cues is strongest for items that bring to mind a large number of clues when searching for the elusive memory target, regardless of the correctness of these clues. These results are consistent with the accessibility model of FOK (Koriat, 1993, 1995), which states that FOK judgments rest on the total amount of partial information accessed in searching for a memory target. However, they also suggest that, as far as the reliance on collectively shared cues is concerned, the accessibility and consensuality of partial information go hand in hand: Reliance on shared clues increases with the total amount of partial clues accessed.

Going back to JOLs, the difference observed between related and unrelated paired associates could lead to the conclusion that the degree of reliance on collective wisdomware depends entirely on the direct, cue-to-target association. Indeed, associative strength—the strength of the cue-to-target association in word association norms—has figured as the main variable affecting JOLs for paired associates in many studies (see Koriat, 1997). The difference in the replicability effect between related and unrelated pairs suggests that word association norms do capture item differences in the collective cues that come to mind in making JOLs for paired associates.

However, the results of Studies 3 and 4 broaden the scope of the type of paired associates that enlist collectively shared cues in making JOLs. These results indicated that not only are JOLs responsive to a posteriori associations and to Backward associations, but also that the cues accessed in responding to these associative relations tend to be collectively shared. Altogether, the results call for extending the examination of the replicability effect for JOLs to other types of paired associates. An important challenge then would be to devise a measure beyond cue-to-target associative strength that captures better the associative relationships that underlie the cross-individual replicability effect across different pairs of words. Such a measure can help specify the kind of relationships between two words that tend to promote reliance on collectively shared cues in making JOLs.

### 4.5. Implications for the Self-Consistency Framework and for the Concept of Collective Wisdom

What are the implications of the results summarized above? In most discussions of the self-consistency model of subjective confidence (e.g., Koriat, 2012, 2018), collective wisdomware was portrayed as a large storehouse of a variety of cues that participants draw on routinely in making their choice and confidence for each binary item. However, the results presented in this article and in Koriat (2026) call for a more refined conceptualization. On the one hand, the results broaden the application of the self-consistency framework to incorporate other forms of metacognitive judgments. On the other hand, these results constrain the applicability of that framework, indicating that it does not apply to all items alike.

These results seem to invite a stronger focus on the process underlying the recruitment of cues when making judgments. A distinction was proposed between the pool of item-specific cues that are available in the memory store for a given item and the sample of cues that is accessed (not necessarily consciously) on the spot in making first-order and second-order judgments (see Koriat et al., 2016). Of course, the cues available place constraints on the cues that are accessed. However, it is the cues that are accessed on a particular occasion that are assumed to affect the actual judgments. Therefore, the question of shared cues pertains to these latter cues, and it is the magnitude of that sharing that is tapped by the cross-individual replicability effect.

The focus on the process underlying the recruitment of cues when making judgments suggests several experimental hypotheses that are worth examination. First, in discussing the measurement of attitudes, it was proposed that because attitudes are formed on the spot, they can vary depending on the context and the person’s goal at the time of forming these attitudes (Schwarz, 2007; Schwarz & Strack, 1991). It is of interest to examine whether the cues recruited in making first-order and second-order judgments also vary depending on contextual factors such as the experimental instructions and the specific learning strategies used by participants in the case of JOLs (see Ingendahl & Undorf, 2026).

Second, the pool of shared cues likely differs to some extent for different groups depending on their backgrounds and social circles (see Galesic et al., 2012, 2018). For example, the words *stroke* and *volley* when presented as a paired associate in a JOL experiment may bring to mind shared cues among tennis fans, yielding a cross-individual replicability effect across them but not across other participants.

### 4.6. The Cue Replication Hypothesis

Reliable inter-item differences may have implications for the cue replication hypothesis proposed by Koriat (2026). Cue replication refers to the overlap between the cues underlying the response to the same item across two different tasks. For example, it refers to the overlap between the cues underlying the study of an item and those underlying the JOL for that item. Similarly, it refers to the overlap between the cues underlying a binary choice and those underlying confidence in that choice.

According to the cue replication hypothesis, the reactivity effect that has been observed for confidence and JOLs (see Double & Birney, 2019; Double et al., 2025; Undorf et al., 2024) should increase with the degree of cue replication. Thus, for example, the reactivity effect for JOLs should be particularly strong when the cues underlying the study of a paired associate are reactivated when making JOLs. This hypothesis has much in common with the cue-strengthening hypothesis (Rivers et al., 2021; Soderstrom et al., 2015).

In Koriat (2026), it was argued that this hypothesis explains why the reactivity effect for JOLs is generally positive for related paired associates but absent or negative for unrelated pairs (Double & Birney, 2019; Rivers et al., 2021; Soderstrom et al., 2015; see Undorf et al., 2024). This argument was based on the results for the cross-individual replicability effect. This effect was found to be strong for related paired associates but weak for the unrelated pairs.

However, Studies 3 and 4 yielded a different pattern of results for cross-individual replicability and within-individual replicability. Whereas the cross-individual replicability effect was not significant for the unrelated pairs, the within-individual replicability effect was significant even for these pairs. These results suggest a revised hypothesis that is worth testing: the reactivity effect should differ depending on the experimental design used. When the JOL solicitation is manipulated between participants, the reactivity effect should be positive for related pairs but negative or weak for unrelated pairs. When it is manipulated within participants, in contrast, the reactivity effect should be consistently positive. Indeed, several discussions (Double & Birney, 2019; Kaya & Mulligan, 2025; Kornell et al., 2009; Metcalfe & Finn, 2008; Myers et al., 2020; Rhodes & Tauber, 2011; Rivers et al., 2021) assumed or implied that the reactivity effect should vary depending on experimental design.

The cue replication hypothesis may also predict the likelihood that participants change their minds when they ponder their choices and decisions. Kosourikhina and Handley (2026) reported that overall cue consistency predicted confidence and response changes.

It was also proposed (Koriat, 2026) that differences in cue replication may explain the observation that JOLs for paired associates do not increase linearly with the strength of the cue-target relationship. Rather, even a weak association is sufficient to yield a strong effect on JOLs. A similar stepwise pattern was observed for the replicability effect. In fact, a similar pattern was also found for the effects of degree of relatedness on semantic priming (Anaki & Henik, 2003; Koriat, 1981), so that differences in the strength of semantic priming may also reflect differences in cue replication.

### 4.7. Final Comments

The results of this study yielded reliable item differences in the cross-individual replicability effect for different forms of metacognitive judgments to the extent that some items hardly evidenced reliance on collectively shared cues. The importance of these item differences is that they provide some clues to the nature of collective wisdomware. They help specify what people who inhabit similar information ecosystems have in common in the ingredients underlying many of their first-order and second-order judgments.

The cross-individual replicability effect provides a useful methodological tool for determining the extent to which different judgments are based on collectively shared cues. A paradoxical feature of that tool is that it relies heavily on between-individual variability to specify what people have in common. The assumption is that this variability is due to the random sampling of cues from the same population of cues (see Fiedler et al., 2023).

Of course, the replicability effect can also be used to uncover differences between different social groups and social cultures (see Galesic et al., 2012, 2018; Koriat & Yehudai, 2026) and also differences between different domains. For example, some of the differences between related and unrelated paired associates in the reactivity effects for JOLs have been examined for English, German, and Hebrew (see Undorf et al., 2024). It is important to extend the range of research to other languages and also to other cultures.

Finally, it should be stressed that the analyses used in this study can be performed on many available datasets involving different forms of metacognitive judgments with no need to collect additional results. Hopefully, the results of these analyses will provide new insights into the notion of collective wisdomware.

## 5. Conclusions

According to the self-consistency framework, participants make their binary choices and metacognitive judgments by drawing a number of cues from collective wisdomware. This study, however, reports results suggesting reliable item differences in the extent to which confidence, feeling of knowing, and JOLs rely on drawing cues from a collectively shared pool of cues. Examination of these differences provides some clues to the nature of the collective wisdomware that is shared by participants with a similar background.

## Figures and Tables

**Table 1:** Mean JOL strength across participants for consensual and nonconsensual JOL-Choice responses for paired associates that differ in degree of associative relatedness (Study 1).

Associative Relatedness	Consensual *M*	Consensual *SD*	Non- Consensual *M*	Non- Consensual *SD*	*t* & *p*	*d*
High	77.8	12.4	58.9	15.2	*t*(15) = 7.06, *p* < 0.001	1.82
Low	75.6	13.1	65.5	14.8	*t*(23) = 5.05, *p* < 0.001	1.05
Unrelated	72.2	14.5	69.5	15	*t*(15) = 0.81, *p* = 0.430	0.21

**Table 2:** The correlations across items between several item properties: mean FOK/Rep, accessibility (ACC), Output-Bound Accuracy (OBA), Percent correct Recognition (RCG), and mean FOK (Study 2, see text).

Item Property	FOK/Rep	ACC	OBA	RCG	Mean FOK
FOK/Rep	1	-	-	-	-
ACC	0.38 ***	1	-	-	-
OBA	0.17	0.23 *	1	-	-
RCG	0.28 **	0.31 **	0.82 ***	1	-
Mean FOK	0.44 ***	0.81 ***	0.32 **	0.42 ***	1

* *p* < 0.05; ** *p* < 0.01; *** *p* < 0.001.

**Table 3:** Mean JOL strength across participants for consensual and nonconsensual JOL-Choice responses for the three classes of items, a priori, a posteriori, and unrelated (Presentation 1, Study 3).

Associative Relatedness	Consensual *M*	Consensual *SD*	Non- Consensual *M*	Non- Consensual *SD*	*t* & *p*	*d*
A priori	82.2	10.5	65.3	14.2	*t*(33) = 7.73, *p* < 0.001	1.35
A posteriori	77.7	11.8	64.6	13.5	*t*(33) = 5.83, *p* < 0.001	1.03
Unrelated	74.9	12.1	68.6	12.8	*t*(25) = 2.88, *p* = 0.008	0.51

**Table 4:** The replicability effect within individuals across Presentations 1 and 2, and the cross-individual replicability effect for each presentation (Study 3, see Text).

**Within-Individual Replicability Across Presentations 1 and 2**	***r***	***t* & *p***
A priori items	0.2	*t*(36) = 3.66, *p* < 0.001
A posteriori Items	0.18	*t*(37) = 5.21, *p* < 0.0001
Unrelated items	0.16	*t*(36) = 3.81, *p* < 0.001
**Cross-Individual Replicability in Presentation 1**	***r***	***t* & *p***
A priori items	0.51	*t*(39) = 11.07, *p* < 0.001
A posteriori Items	0.42	*t*(39) = 7.76, *p* < 0.001
Unrelated items	0.07	*t*(39) = 1.54, *p* = 0.131
**Cross-Individual Replicability in Presentation 2**	***r***	***t* & *p***
A priori items	0.53	*t*(39) = 11.42, *p* < 0.001
A posteriori Items	0.43	*t*(39) = 8.52, *p* < 0.001
Unrelated items	0.06	*t*(39) = 0.97, *p* = 0.338

**Table 5:** Mean JOL strength across participants for consensual and nonconsensual JOL-Choice responses for the three classes of items, Forward, Backward, and Unrelated in Presentation 1 (Study 4).

Associative Relatedness	Consensual *M*	Consensual *SD*	Non- Consensual *M*	Non- Consensual *SD*	*t* & *p*	*d*
Forward	82.6	10.1	58.4	14.5	*t*(24) = 10.15, *p* < 0.001	2.03
Backward	82.3	11.2	64.7	13	*t*(35) = 9.30, *p* < 0.001	1.55
Unrelated	75.8	13.4	76.1	14.1	*t*(30) = 0.10, *p* = 0.921	0.02

**Table 6:** The replicability effect within individuals across presentations 1 and 2, and the cross-individual replicability effect for each presentation (Study 4, see Text).

**Within-Individual Replicability Across Presentations 1 and 2**	***r***	***t* & *p***
Forward items	0.33	*t*(30) = 5.16, *p* < 0.001
Backward Items	0.3	*t*(34) = 4.83, *p* < 0.001
Unrelated items	0.19	*t*(38) = 4.49, *p* < 0.001
**Cross-Individual Replicability in Presentation 1**	***r***	***t* & *p***
Forward items	0.52	*t*(38) = 8.47, *p* < 0.001
Backward Items	0.49	*t*(39) = 9.17, *p* < 0.001
Unrelated items	0.04	*t*(39) = 0.65, *p* = 0.519
**Cross-Individual Replicability in Presentation 2**	***r***	***t* & *p***
Forward items	0.65	*t*(37) = 9.42, *p* < 0.001
Backward Items	0.58	*t*(39) = 9.91, *p* < 0.001
Unrelated items	0	*t*(39) = 0.08, *p* = 0.937

**Table 7:** The replicability effect within individuals across Presentations 1 and 2, and the cross-individual replicability effect for each presentation (Study 5, see Text).

**Within-Individual Replicability Across Presentations 1 and 2**	***r***	***t* & *p***
Lines task	0.18	*t*(38) = 6.01, *p* < 0.001
Shapes task	0.21	*t*(40) = 8.39, *p* < 0.001
**Cross-Individual Replicability in Presentation 1**	***r***	***t* & *p***
Lines task	0.28	*t*(38) = 9.52, *p* < 0.001
Shapes task	0.35	*t*(40) = 14.74, *p* < 0.001
**Cross-Individual Replicability in Presentation 2**	***r***	***t* & *p***
Lines task	0.24	*t*(38) = 7.61, *p* < 0.001
Shapes task	0.39	*t*(40) = 13.42, *p* < 0.001

## Data Availability

The data presented in this study are available on request from the corresponding author.
